# Development of the Dutch translational knowledge agenda for inherited metabolic diseases

**DOI:** 10.1002/jmd2.12455

**Published:** 2024-12-22

**Authors:** I. J. Hieltjes, J. H. van der Lee, M. C. Groenendijk, G. van Haaften, P. M. van Hasselt, R. J. Lunsing, G. J. J. van Prooijen, E. M. de Ruiter, F. J. van Spronsen, N. M. Verhoeven‐Duif, A. de Vreugd, M. Wagenmakers, H. Zweers, H. Dekker, H. R. Waterham, C. D. van Karnebeek, R. J. A. Wanders, R. A. Wevers

**Affiliations:** ^1^ Knowledge Institute of the Dutch Association of Medical Specialists Utrecht The Netherlands; ^2^ Department of Pediatrics and Human Genetics, Emma Center for Personalized Medicine Amsterdam UMC Amsterdam The Netherlands; ^3^ MetaPACT The Netherlands; ^4^ Stichting Stofwisselkracht The Netherlands; ^5^ United for Metabolic Diseases (UMD) The Netherlands; ^6^ Department of Genetics UMC Utrecht Utrecht The Netherlands; ^7^ Department of Metabolic Diseases, Wilhelmina Children's Hospital UMC Utrecht Utrecht The Netherlands; ^8^ Department of Pediatric Neurology, University Medical Center Groningen University of Groningen Groningen The Netherlands; ^9^ Patiëntenvereniging voor Stofwisselingsziekten (VKS) The Netherlands; ^10^ Department of Pediatrics Radboud University Medical Center Amalia Children's Hospital Nijmegen The Netherlands; ^11^ Department of Internal Medicine, Erasmus MC, Center for Lysosomal and Metabolic Disease Erasmus University Medical Center Rotterdam Rotterdam The Netherlands; ^12^ Diëtisten Erfelijke Stofwisselingsziekten (DIES); ^13^ Laboratory Genetic Metabolic Diseases, Department of Laboratory Medicine Amsterdam UMC Amsterdam The Netherlands; ^14^ Department of Human Genetics, Translational Metabolic Laboratory (TML) Radboud University Medical Center Nijmegen The Netherlands

**Keywords:** gene therapy, knowledge agenda, metabolomics, natural course, pathophysiology, patient and public involvement, personalized medicine, prevention, psychosocial burden, quality of life, rare diseases, research priorities, trial design

## Abstract

**Background:**

Inherited metabolic diseases (IMDs) may have considerable implications for patients and their families. Despite their individual rarity, covering a spectrum of over 1800 distinct diseases, the diseases collectively exert a significant impact, with often lifelong disabilities. The United for Metabolic Diseases consortium was established to catalyze research with translation into the best possible care.

**Aim:**

To generate a translational knowledge agenda, which identifies and prioritizes research questions, directly relevant to patient care or for IMD patients and their families.

**Methods and Results:**

Following a process established by the Knowledge Institute of the Dutch Association of Medical Specialists, we generated a comprehensive translational knowledge agenda for IMDs. A multidisciplinary steering committee, composed of 12 diverse metabolic experts collected research questions through an online questionnaire using snowballing. The 462 proposed questions were categorized and prioritized during a meeting attended by 22 representatives of all stakeholder groups. The resulting top 10 research questions cover multiple themes, i.e. prediction of disease progression, development of novel tools, mechanistic insights, improved diagnostics, therapeutic integration of multi‐omics techniques, assessment of impact on daily life, expanding treatment avenues, optimal study designs, effect of lifestyle interventions, and data utilization using FAIR principles.

**Discussion:**

This collective endeavor reflects the collaborative spirit needed for rare disease research. This knowledge agenda will guide funding directions and applications but will also boost interdisciplinary collaboration to push the field of IMDs research forward in a renewed UMD consortium. Patient engagement, transparency, and a comprehensive approach make this knowledge agenda a pivotal step toward addressing the pressing research needs and priorities in this domain.

**Synopsis**

Translational knowledge agenda for Inherited metabolic diseases.

## 1 INTRODUCTION

Inherited metabolic diseases (IMDs) have a profound impact on the lives of patients and their families. The prevalence of these disorders varies across different populations, with estimates ranging from 1:318 to 1:3760 live births.^1^ IMDs encompass a wide range of over 1800 different diseases that can manifest at any stage of life.^2^ While individually these diseases may be rare, collectively they represent a significant cause of morbidity and mortality as well as personal and societal stress.

Most IMDs are autosomal recessive. Biochemical pathways and processes are disturbed, which may result in a shortage of energy, a lack of building blocks for cells and tissues, or a buildup of toxic metabolites. All organs can be affected. The clinical presentation of IMDs is highly diverse even within a single disease entity. This often leads to a considerable diagnostic delay and requires the involvement of multiple clinical disciplines in the diagnostic and management process. The IMDs form the largest group of treatable conditions. While for some IMDs effective treatment options are available, many of these conditions still lack specific therapeutic interventions.^3^

Dutch researchers and specialists have always been collaborative in the research on IMDs. In 2018, this collaboration was formalized through the establishment of United for Metabolic Diseases (UMD),^4^ which aims for a healthier future for all metabolic patients and their families via innovation and acceleration of diagnosis, treatment, care, and prevention. UMD is a national consortium comprising internationally recognized experts in metabolic diseases from six Dutch University Medical Centers, as well as patient representatives represented by the Dutch patient organization ‘Volwassenen en Kinderen, met Stofwisselingsziekten’ (VKS – “Adults and children with inherited metabolic diseases”). Furthermore, in 2022 more than 30 national patient organizations for IMDs united in MetaPACT (METAbolic Patients Advocacy, Care and Treatment).

The development of a comprehensive national translational knowledge agenda for metabolic diseases was considered crucial to make optimal use of improvements in diagnosis and therapy thus improving patient outcomes. Patient and public involvement are increasingly recognized as essential for the quality, relevance, and credibility of clinical research. To this end, patients, carers, and healthcare professionals should collaborate to establish research priorities for the field.^5^ This formed the basis of this first knowledge agenda for metabolic diseases in the Netherlands.

During the past decade, the Knowledge Institute of the Dutch Association of Medical Specialists has supported the development of over 40 knowledge agendas for medical specialists' associations. These knowledge agendas aimed to prioritize research topics in the field of clinical care evaluation and to decrease translational gaps between evidence from clinical research and recommendations in guidelines. A central principle in the development of these knowledge agendas is the involvement of all stakeholders in the inventory and prioritization of knowledge gaps and research questions.^6^ For IMDs, the lack of knowledge is of such a substantial nature that it exceeds the idea of ‘knowledge gaps’; therefore, the preferable term for the items on the translational knowledge agenda is ‘research questions’.

In the field of IMDs, there are several examples in the literature of international research agendas that were developed for one specific disease or group of diseases, e.g. nephropathic cystinosis, liver glycogen storage disease, and mitochondrial disorders.^7^, ^8^, ^9^ The methodology used in these projects is similar to the Dutch knowledge agendas.

In 2022, UMD and the Knowledge Institute of the Dutch Association of Medical Specialists decided to collaborate with the aim of generating a translational knowledge agenda for IMDs. The main question was: what are the most urgent research questions in the field of IMDs that need to be addressed on a national level in the Netherlands in the coming 4–8 years?

## 2 METHODS

For the development of the knowledge agenda, the general approach of the Knowledge Institute was followed.^6^ A steering committee was installed consisting of 12 experts representing laboratory specialists, metabolic researchers, metabolic pediatricians, internists, pediatric neurologists, dieticians, nurse specialists, and patient organizations.

First, an inventory was made of all possible research questions, involving a broad group of stakeholders. Research questions were collected in three ways; (1) research questions were extracted from a 2019 policy document of UMD, (2) the results of a survey conducted by the VKS in 2020 were searched for research questions, and (3) an e‐mail invitation for an online survey was distributed by snowballing to various groups including healthcare professionals, researchers, and patient organizations. The main question of the online survey was: ‘Which research questions should be included in the knowledge agenda for inherited metabolic diseases in your opinion?’. A separate layman explanation was added for non‐professionals. All members of the steering group invited their colleagues to fill in the survey and distributed the link to potentially interested contacts. Two reminders were sent in June and July 2022.

After the collection phase, duplicate research questions were removed or combined. The resulting set of research questions was reviewed by two members of the steering group, independently. Reasons to exclude individual entries were: (a) research questions unrelated to IMDs, (b) if considered too vague or not specifically formulated, (c) when not addressing a knowledge gap or research question, (d) if political and/or organizational in nature, or (e) when already known to be addressed in ongoing research projects. In instances where uncertainty arose regarding a research question (for instance around feasibility), it was presented for discussion within the steering group. The steering group members subsequently discussed discrepancies in the assessment until consensus was reached.

All remaining research questions were categorized into eight themes: (1) primary prevention of disease and secondary prevention of disease symptoms, (2) diagnostics – improved diagnostic strategies, (3) disease severity measures and disease progression measures, (4) therapy, care, and outcomes, (5) nutrition, (6) disease mechanisms and disease models, (7) cost‐effectiveness, and (8) knowledge sharing and databases.

A full‐day prioritization meeting took place on November 1, 2022. Twenty‐four participants from different stakeholder groups were personally invited. The composition of this group aimed to do justice to the number of experts in the field of IMDs of each discipline. The group consisted of equal numbers of patient representatives and professionals. The research questions were divided into five groups, which were discussed at five different tables. The eight aforementioned themes were divided over the five tables. The number of research questions per table was comparable (see Appendix S1). The twenty‐four participants were invited in advance to indicate one or more of their preferred themes. Patient representatives were assigned to the table of their preference; experts were assigned to the tables at random during the first round of the meeting, and to the table of their preference during the second round. At each table, members of the steering group chaired the discussion. The steering group members did not have a voting right and chaired the same table in both rounds.

Before the start of the meeting, prioritization criteria had been developed by the steering group for all research questions discussed during the prioritization meeting.

The prioritization criteria were as follows:

- *Patient‐centered*. The questions should involve translational research, with a clear connection to patients with an IMD. For this reason, fundamental research questions or investigations into metabolic processes were excluded if it was not evident beforehand whether IMD patients ultimately would benefit from it.
- *Implications for the development of the entire field*. Research questions should be of broad general interest to a (larger) number of IMDs, either directly or indirectly. This includes broad applicability and scientific and/or societal relevance.
- *Unmet need*. Research questions that can make a significant difference in the lives of patients with an IMD are given higher priority on the agenda especially when the specific research question(s) concerned (preferably) several IMDs.
- *Feasibility of research (within 4–8 years)*. High on the agenda are research questions that can yield answers within a period of up to 4–8 years. If a longer period is required to obtain valuable results, the likelihood of securing funding decreases. The further the horizon, the lower the priority on the agenda.
- *Relevance for other stakeholders*. The knowledge agenda will have a greater impact when the highly prioritized research questions are not only relevant to a specific group (e.g., researchers in a particular metabolic field) but also to a broader range of stakeholders, such as the government, Ministry of Health, Welfare and Sport (VWS), Netherlands Organization for Scientific Research (NWO), Netherlands Organization for Health Research and Development (ZonMw), Insurance companies, and Patient advocacy organizations.

In round one of the prioritization meeting, the number of research questions per table was reduced to a top 10. In round two, the top 10 from the first round was reviewed and could be adjusted if deemed necessary. The “one in, one out” principle was applied, and changes had to be motivated. The aim of the second round was ultimately to go from a top 10 to a top 5 per table. Round three was a plenary round, in which the top 5 per table was explained to all participants by the table chairs and overlap in the 25 research questions was discussed. In round 4, the participants were asked to indicate their personal top 5 through voting. After the meeting, the votes were analyzed by counting the number of votes per research question. These results were discussed by the steering group in a separate meeting to establish the final top 10.

## 3 RESULTS

In total 158 respondents completed the online survey. Table 1 shows the backgrounds of the respondents.

**TABLE 1:** Overview of the respondents and participants.

Background	Respondents' questionnaire *N* (%)	Participants prioritization meeting *N* (%)
Patient representatives	85 (54)	8 (36)
Pediatricians	17 (11)	3 (14)
Internists	6 (4)	2 (9)
Pediatric neurologists	6 (4)	1 (5)
Researchers	14 (9)	2 (9)
Laboratory specialists	13 (8)	3 (14)
Dieticians	9 (6)	2 (9)
Nurse specialists	4 (3)	1 (5)
Other/unknown	4 (3)	–

**FIGURE 1:**
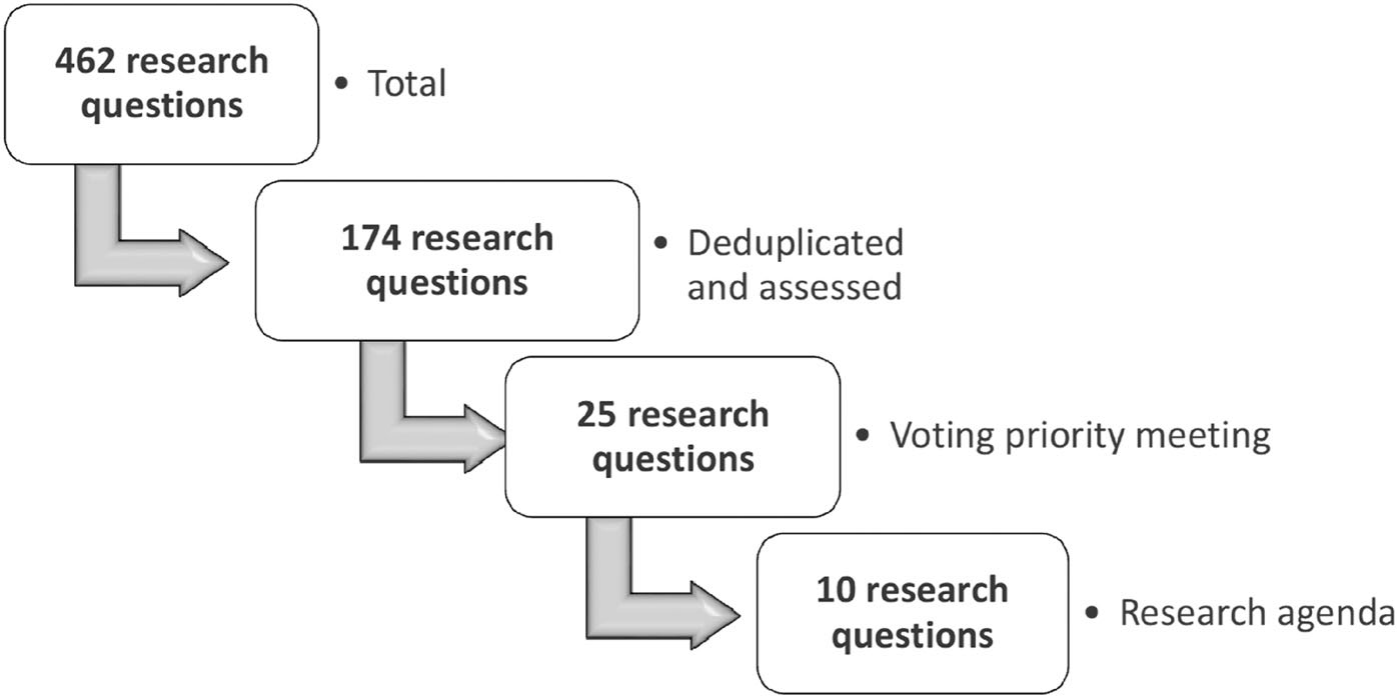
Flowchart of the research questions per round.

Figure 1 shows the numbers of research questions per round in a flowchart. The total number of research questions was 462, of which 37 originated from the UMD policy document and the earlier VKS survey and 425 from the online survey. After deduplication and selection by the steering group, 174 research questions remained that were divided over the eight themes, which formed the basis of the five discussion tables for the prioritization meeting. Each person invited to the prioritization meeting received the research questions divided per discussion table and the prioritization criteria with an explanation of the prioritization meeting goals. Two invited persons were absent on the day of the prioritization meeting, which implied that there were 22 participants in addition to the steering group members, eight patient representatives, three pediatricians, two internists, one pediatric neurologist, two researchers, three laboratory specialists, two dieticians and one nurse specialist (Table 1). During the prioritization meeting, a selection of 25 research questions was made. Subsequently, participants, excluding steering group members, used voting forms to select their top 5 questions. During the meeting, there was a discussion about whether this voting should be anonymous. According to the methodology usually used by the Knowledge Institute, the voting would be open, by sticking notes on posters.^6^ However, after some discussion, it was unanimously decided by the participants that the voting should be anonymous to preclude that participants would be influenced by the votes of others. After analysis of the voting and discussion with the steering committee, the 10 most relevant research questions were defined.

The 10 research questions selected for the knowledge agenda with accompanying context are the following, in random order:

### 3.1 How can we monitor and predict the expected disease progression with and without intervention?

Understanding and predicting the course of IMDs is challenging. Challenges include the rarity of each of the IMDs, variations in the severity of gene mutations, the multi‐systemic character of clinical symptoms, the disease course, and the timing and efficacy of interventions. The progressive nature of many of the IMDs requires age‐contextualized interpretation. The insufficiently known disease trajectories and the limited tools for mapping them necessitate new assessment tools. Developing a versatile toolbox to capture disease‐specific characteristics is a key challenge. Identifying “disease trajectory markers” that can be used for standardized detection of relevant changes is crucial. Disease progression assessment requires insights across metabolites, cellular, physical, cognitive, and behavioral domains. The suitability of existing biomarkers and potential novel biomarkers, including metabolomics and RNA‐sequencing, should be explored. Patient Reported Outcome and Experience Measures (PROMs and PREMs), along with emerging technologies, hold promise in data collection for comprehensive disease understanding and management.

### 3.2 Which tools need to be developed to describe the disease course in detail and determine the effects of treatments?

In the development of assessment tools, consideration must be given to the patient's life stage based on the notion that the course of disease varies with age as well as overall heterogeneity. Information will emerge from various domains (including PREMs, clinical data, and biomarkers) and meaningful integration is necessary. Interpretation of complex data requires specialized statistical analysis software, potentially necessitating further development. Artificial intelligence software could potentially play a role in this context. The harmonization of diverse data streams requires advanced techniques to extract valuable insights for personalized patient care and disease management. The incorporation of patient‐reported outcomes, clinical assessments, and biomarkers will contribute to a comprehensive understanding of disease progression and treatment efficacy.

### 3.3 What are the underlying mechanisms in IMDs?

Understanding the intricate mechanisms underlying IMDs is of crucial importance in several aspects including the generation of innovative therapies and providing explanations for the clinical diversity among patients, potentially influenced by non‐genetic factors. Currently, determinants governing disease severity and progression are largely unknown. Conventional research has often focused on single genetic defects, thereby ignoring the fact that proteins are embedded in large networks and do not operate in isolation. A systems biology approach is necessary to grasp modifying mechanisms comprehensively. This perspective is pivotal for developing effective therapeutic strategies. Recent advancements in omics techniques and sophisticated models provide this perspective, unveiling common pathophysiological mechanisms across IMDs. Collaboration between clinical and fundamental scientists is essential to drive novel treatments based on improved pathophysiological understanding.

### 3.4 How can screening be utilized to minimize the (often lifelong) disease burden among newborns as much as possible?

Two strategies are envisioned to substantially alleviate the long‐term disease burden among newborns through population‐based screening programs. The first involves expanding the widely accepted newborn bloodspot screening for treatable conditions. The current screening covers a limited number of diseases. Introducing DNA techniques into this screening could potentially add numerous treatable diseases. Nevertheless, comprehensive research addressing practical, ethical, and societal concerns is essential for successful implementation.

The second strategy involves preconception carrier screening, which involves genetic screening of both prospective parents for hundreds of recessive genetic diseases. If both parents are carriers of a defect in the same gene, they receive options to prevent the birth of a child with a severe disease. Although practiced in various countries, preconception carrier screening faces ethical, logistical, economic, and implementation‐related challenges before being considered in the country.

The inclusion of “actionable” yet untreatable diseases in the current newborn bloodspot screening is discouraged because of potential implications for the present participation rate. of newborn screening for treatable diseases only. Instead, an opt‐in program at consultation centers might offer opportunities for an early diagnosis. A thorough evaluation of advantages, disadvantages, and ethical dimensions is essential before contemplating implementation.

### 3.5 Can we improve the diagnostics, therapy, and follow‐up of IMDs through the integration of DNA, RNA, and omics techniques?

The diagnostics of IMDs are significantly advancing, thanks to key developments in sequencing of DNA (whole exome and whole genome sequencing, respectively WES and WGS) and RNA along with the implementation of other omics techniques. Untargeted omics techniques, including metabolomics, lipidomics, and glycomics, will complement or replace traditional targeted analyses and provide a functional read‐out of genetic variants. Integrating DNA, RNA, and omics data could lead to substantial progress in diagnostics and will greatly aid in interpreting the pathophysiological consequences of the many variants of uncertain significance (VUS) detected by DNA sequencing. However, challenges in turnaround time and identification of unidentified metabolites need to be addressed for optimal use. Next to their diagnostic potential, omics techniques allow the identification of new biomarkers for prediction and monitoring of response to therapeutic interventions and may offer a more comprehensive understanding of metabolic disruptions resulting from an enzyme or transporter defect.

### 3.6 What is the impact of IMDs on daily life, how do we assess this impact, and how do we implement this knowledge in healthcare?

After the initial presentation of an IMD, it significantly impacts both the patient and their family. The continuous visits to doctors and hospitals, frequent examinations, prolonged uncertainty about the disease progression, therapeutic measures, lifestyle adjustments, financial implications, and sometimes specialized dietary strategies collectively impose substantial physical, mental, and psychosocial burdens. Finding guidance for living with such a condition is crucial. Patient Reported Experience Measures (PREMs) and Patient Reported Outcome Measures (PROMs) could potentially facilitate shared experiences and knowledge among those in similar circumstances. Patient associations and support groups, often connected via social media or the internet, play a vital role. Tailored advice and interventions are needed, considering factors like age, disease type, and transitional care. Research on enhancing patients' and families' psychosocial well‐being and the role of lifestyle is necessary. Tailored quality of life‐focused assessment tools are vital for evaluating treatment benefits, considering the balance between treatment effectiveness, the patient's baseline, and expected outcomes without treatment. This research aims to support optimal patient functioning while minimizing unnecessary hospitalization or medicalization.

### 3.7 How can therapeutic approaches be developed for the large group of IMDs that currently lack available treatments?

Of the over 1800 IMDs, currently less than 20% have one or more effective and available treatment options (e.g. dietary therapies or enzyme replacement therapies). This emphasizes the need for new, safe, and effective treatment options. For some IMDs repurposing of drugs, which are already available for different indications, may be a good option. Furthermore, research on stem cell differentiation and different forms of genetic therapies (including for example ex‐vivo hematopoietic stem cell gene therapy, RNA‐ based therapies and oligonucleotide therapies), show great promise. Important steps have already been taken, which are vital for new therapies to become available. For different forms of gene therapy, safety and efficacy has already been validated. However, great challenges remain, also regarding efficiency. Research to identify the most optimal form of genetic therapy, in all aspects, is crucial. The therapies should always reach the target organ or cell type. Techniques to accomplish this need to be improved. In an increasing number of IMDs multiple innovative treatments are being developed for the same rare disease, raising complicated issues regarding prioritization. Another point of concern is the development and implementation costs of new therapies making it impossible to develop therapies for all IMDs at the same time: Which diseases should be given priority? To address these issues active involvement of patient organizations is vital. To maximize cost‐effectiveness and speed up clinical applications, considering streamlining therapy development processes across diseases could be beneficial.

### 3.8 Which clinical study designs are suitable, and what requirements need to be met, to ensure access to a new therapy for patients with an IMD?

Evaluating treatment efficacy and safety is preferably done in large patient groups using randomized placebo‐controlled trials, but this is not possible in ultrarare diseases. Furthermore, for extremely rare diseases, finding cost‐effective treatment solutions is challenging. Research to investigate adequate trial methods for the evidence‐based evaluation of the safety and efficacy of new therapies in multiple rare diseases is crucial, aiding efficient therapy development. For rare diseases, N‐of‐1 trials as well as other innovative designs may offer solutions. Alternative trial designs like sequential/adaptive designs, which are not yet used in IMDs but were of great values during the COVID pandemic, need to be explored. This may aid the development of models that assist larger numbers of patients with many different IMDs in a safe, efficient, and affordable manner. Vital aspects include trial design and criteria for assessing efficacy and safety across various outcome levels (biological, physiological, functional, quality of life and participation). These elements are pivotal for patients, researchers, and clinicians Moreover, establishing the criteria for trial design in rare diseases is needed to convince regulatory and reimbursement agencies.

### 3.9 How can lifestyle and nutrition influence the natural course of IMDs and improve quality of life?

Many IMDs can be treated well with specific diets, and a healthy lifestyle improves health. A comprehensive lifestyle intervention, including diet guidance, exercise, and psychological support, is now used for obesity. Common symptoms in these conditions can potentially be improved through diet, lifestyle advice, and rehabilitation. Consequently, patients and healthcare providers inquire about optimizing these factors, often due to a lack of scientific evidence for specific conditions. The research focuses on identifying diseases that can benefit and determining how natural disease progression can be positively influenced. Understanding how to implement optimal nutrition and appropriate exercise is crucial. Studies in conditions like Pompe disease (Online Mendelian Inheritance in Man, MIM#606800) and mitochondrial disorders are ongoing. It's important to explore the disease‐modulating potential and consider both dietary elements and movement patterns to be avoided. Assessing the impact on patients' quality of life and balancing benefits with drawbacks are vital.

### 3.10 What is the best way to utilize research/patient data according to the FAIR (Findable, Accessible, Interoperable, Reusable) principles for research on IMDs?

In investigating the natural course or effects of interventions, systematic collection of real‐world data from as many patients as possible over extended periods is crucial. This demands intensive involvement of patients and families, along with international collaboration due to the rarity of most IMDs. In addition to clinical and genetic‐metabolic laboratory data, inclusion of imaging and general clinical chemistry information is recommended. The current era of digital data offers opportunities, but data collection is challenged by complex IT systems and increasing regulations like the European and national General Data Protection Regulation (GDPR). Compliance with the FAIR Guiding Principles^10^ is essential for optimal usability for research purposes. Several outdated multicenter data collection systems exist for these diseases, and initiatives like Metab‐ERN, Dutch Diagnoss Registration Metabolic Diseases (DDRMD) and the Unified European Registry for Inherited Metabolic Disorders aim to integrate new data collection systems with old ones. However, these systems may underutilize advanced data collection and AI techniques for data interpretation, which could significantly enhance research efficiency and effectiveness for rare IMDs.

In addition to the 10 prioritized research questions above, the topic ‘guidance and monitoring of pregnancy in IMDs’ was considered important. However, it was decided not to include this in the knowledge agenda as it is not currently feasible to set this up at the national level because there are not enough cases. It is expected, however, that progress on this topic will be gained through international collaboration.

## 4 DISCUSSION

The aim of this knowledge agenda was to define and prioritize research questions that are directly relevant to patient care for IMD patients and families, with the ultimate aim of achieving improved care and outcomes for affected individuals.

Unique aspects of this knowledge agenda include the focus on translational research, ‘from bench to bedside,’ the nationwide involvement of UMD's care and research professions as well as 30 patient organizations united in MetaPACT, and the broad collection of rare disorders being considered.

Throughout its development, patients' input and perspectives were consistently incorporated at each stage. This level of patient engagement corresponds to the highest level of the patient participation ladder,^11^ signifying equal partnership.

The active patient involvement ensured that lifestyle‐related questions and psychosocial burdens were identified and included as critical aspects from the patients' perspective. Subsequently, these questions were grouped into a comprehensive research question under the theme “lifestyle”.

During the prioritization meeting, participants raised concerns regarding the usual voting method, which is non‐anonymous and involves the use of stickers. This was seen as a risk of influencing the voting by other participants. As a result, a last‐minute change was implemented to ensure that every participant could vote freely in line with the participants' strong commitment to develop an unbiased knowledge agenda. One of the reasons why anonymity may have been a more sensitive issue in this prioritization process than in other, more ‘monodisciplinary’ knowledge agendas, was the background variety of the participants. This change in method distinguishes this knowledge agenda from other, earlier knowledge agendas made by the Knowledge Institute and within the field of IMDs.^6^, ^8^

A limitation of this knowledge agenda may be that the broad and translational nature of the field made it very challenging to prioritize only 10 research questions. Additionally, some participants with a particular interest in a specific rare disease might find their own research questions not explicitly mentioned in the knowledge agenda, potentially impacting their sense of connection to the knowledge agenda. Nevertheless, the collaborative nature of this endeavor remains crucial, as research on rare diseases necessitates collective efforts, transcending individual initiatives. The steering group has made considerable effort to maintain optimal transparency so that every decision was captured.

The snowballing method employed during the inventory phase has its limitations, as it does not permit a response rate calculation and precludes the identification of any potential missing respondent group(s). Despite this drawback, snowballing does offer the advantage of reaching numerous participants in an efficient way.

This knowledge agenda provides a strong basis to apply for funding from national funding organizations. Furthermore, this knowledge agenda has the potential to foster interdisciplinary collaboration, providing a clear research focus for the forthcoming years. Patient empowerment within this knowledge agenda is obvious, as patient organizations actively participated in the development process.

In conclusion, this translational knowledge agenda for IMDs covers the top 10 prioritized research questions, setting the course for scientific research in the coming 4–8 years for United for Metabolic Diseases and beyond.

## CONFLICT OF INTEREST STATEMENT

The authors have no competing interests to declare.

## ETHICS STATEMENT

This translational knowledge agenda does not contain any work on humans or animals.

## Supporting information


Appendix S1.

## Data Availability

The data that support the findings of this study are available from the corresponding author upon reasonable request.
